# Dynamics of Interleukin-9 Producing Lymphocytes in *Strongyloides ratti*-Infected Mice

**DOI:** 10.3390/pathogens15030257

**Published:** 2026-02-28

**Authors:** Wiebke Hartmann, Lennart Heepmann, Lara Linnemann, Paula Licona-Limon, Florent Colomb, Tania Frangova, Henry J. McSorley, Minka Breloer

**Affiliations:** 1 Helminth Immunology, Section Interface, Bernhard Nocht Institute for Tropical Medicine, 20359 Hamburg, Germany; hartmann@bnitm.de (W.H.); lennart.heepmann@bnitm.de (L.H.); lara.linnemann@bnitm.de (L.L.); 2 Departamento de Biología Celular y del Desarrollo, Instituto de Fisiología Celular, Universidad Nacional Autónoma de México, Mexico City 04510, Mexico; plicona@ifc.unam.mx; 3 Faculty of Life Sciences, University of Dundee, Dundee DD1 5EH, UKhmcsorley001@dundee.ac.uk (H.J.M.); 4 Department of Biology, University Hamburg, 22609 Hamburg, Germany

**Keywords:** IL-9, ILC2, *Strongyloides*, immunosuppression, CD4^+^ T cells

## Abstract

Helminths infect a quarter of the human population and are controlled in the frame of a canonical type-2 immune response. Interleukin-9 is a cytokine with pleiotropic functions during type-2 immunity that can be produced by many different cells. Accumulating evidence suggest that IL-9 is of particular relevance in controlling intestinal helminth infections. Using mice infected with the parasitic nematode *Strongyloides ratti*, we showed previously that ejection from the intestine depends on IL-9 and IL-9-mediated activation of mucosal mast cells. Here we use IL-9 reporter mice to identify the relevant cellular sources of IL-9 in vivo. We report that predominantly CD4^+^ T cells and group 2 innate lymphoid cells (ILC2s) produced IL-9 in *S. ratti*-infected or IL-33-treated mice. Interestingly, the IL-33-mediated induction of IL-9 and subsequent mast cell degranulation was modulated by concurrent *S. ratti* infection. While the IL-33-mediated expansion of IL-9-producing ILC2s was supressed by *S. ratti* infection, IL-9-producing CD4^+^ T cells were proportionally increased. Finally, we show that *S. ratti*-derived E/S products interfered with IL-9 production by BM-derived ILC2 in vitro. In conclusion, we have identified that ILC2 and CD4^+^ T cells produce IL-9 during *S. ratti* infection, and that ILC2 responses are suppressed by *S. ratti* products.

## 1. Introduction

A quarter of the human population is currently infected with soil transmitted helminths [1], of which the parasitic nematode *Strongyloides stercoralis* accounts for approximately 600 million cases worldwide [2]. The rodent-specific *Strongyloides ratti* can be used to study the protective immune response, as well as parasite–host crosstalk, in the mouse system [3]. The *Strongyloides* life cycle consists of free-living and parasitic stages [4,5], where infective third stage larvae (L3) live in the free world and actively penetrate the skin of their host, migrate through tissue for the following 48 h and are found in the small intestine (SI) by day 3 post-infection (p.i.). In the intestine, larvae moult to parasitic adults that live embedded in the intestinal mucosa and reproduce via parthenogenesis starting from day 5 or 6 p.i. Eggs and first-stage larvae (L1) are released with the feces and may either directly develop into L3 or undergo one free-living generation. *Strongyloides* infection elicits a canonical type 2 immune response in infected rats and mice, resulting in parasite clearance after 2–4 weeks (reviewed in [3]). Approximately 90% of the tissue-migrating L3 are opsonized by complement [6] and antibodies [7,8] and subsequently killed by eosinophils and neutrophils [9]. Ejection from the intestine is promoted by basophils [10] and mucosal mast cells [11] in an IL-9-dependent manner [12].

IL-9 is a cytokine with pleiotropic function that can be produced by a plethora of cells such as T cells, mast cells, eosinophilic, basophilic and neutrophilic granulocytes, as well as group 2 innate lymphoid cells (ILC2s) [13,14]. Accumulating evidence suggests that IL-9 also promotes the ejection of other intestinal helminth parasites including *Trichuris muris* [15,16,17] and *Trichinella spiralis* [18,19,20]. The importance of IL-9 during *Nippostrongylus brasiliensis* infection, a common mouse model for human hookworm infections, apparently depends on the genetic background of the mice. IL-9-deficient 129 × C57BL/6 (F_2_) mice had a similar intestinal parasite burden as the WT controls despite impaired mast cell activation [21]. IL-9-deficiency on a BALB/c background, by contrast, increased *N. brasiliensis* parasite burden in the context of impaired expansion of eosinophils, basophils and mast cells [22]. Likewise, IL-9 receptor knockout (IL-9R KO) mice showed a delayed clearance of *N. brasiliensis* from the SI [23]. Regarding *S. ratti*, we demonstrated previously that IL-9R KO mice on both BALB/c and C57BL/6 genetic backgrounds had elevated intestinal parasite burden and reduced mucosal mast cell degranulation during *S. ratti* infection [12]. Furthermore, neutralization of endogenous IL-9 via application of a monoclonal Ab elevated intestinal *S. ratti* parasite burden in the context of reduced mast cell degranulation, while i.p. injection of recombinant IL-9 reciprocally reduced intestinal parasite burden in wildtype BALB/c and C57BL/6 mice [24]. Finally, we observed that IL-9-mediated mast cell activation was actively antagonized by *S. ratti* via the expansion of Treg and the induction of negative regulatory receptors on T cells in vivo [24,25].

While these findings establish a central role for IL-9 in protective immunity to most intestinal helminth infections, the relevant cellular sources of IL-9 are still understudied (reviewed in [26]). Development of IL-9 reporter and IL-9 fate reporter mouse models revealed both CD4^+^ T cells and ILC2s as important IL-9-producing cell populations in *N. brasiliensis* infection [22,23,27].

Here we use IL-9 reporter mice to characterize the dynamics of IL-9-producing cell populations during *S. ratti* infection. We record increased frequencies of IL-9-producing cells in the mesenteric lymph nodes (mLNs) and SI by day 10 of infection and identify T cells and ILC2s as important IL-9 producers. When IL-9 production is provoked by IL-33-treatment, we observe downmodulation of IL-9-producing ILC2 frequencies by concurrent *S. ratti* infection in vivo and demonstrate reduction in IL-33-induced IL-9 production by *S. ratti*-derived E/S products in vitro.

## 2. Materials and Methods

### 2.1. Mice and Rats

All animal experiments were performed at the animal facility of the Bernhard Nocht Institute for Tropical Medicine (BNITM) in accordance with the German Animal Welfare Act and approved by the relevant German authority (Behörde für Gesundheit und Verbraucherschutz, Hamburg, Germany) under the following approval numbers: N01/2021 and A20/2020. Wistar rats and INFER mice [22] were bred at the BNITM. BALB/c and C57BL/6 mice were purchased from Charles River. Animals were housed under specific-pathogen-free conditions in individually ventilated cages, with a maximum of five mice or two sex-matched rats per cage.

### 2.2. Parasites and Infections

The life cycle of *S. ratti* was maintained at the BNITM by infection of male and female Wistar rats via s.c. injection of 2500 L3 as described [28]. Briefly, feces of infected rats were collected at day 7 and incubated for 7 days with charcoal at 25 °C and 100% humidity. Infective *S. ratti* L3 were isolated from the cultures via the Baermann apparatus as described [28]. The larvae were washed twice with 1× PBS supplemented with penicillin/streptomycin. To infect mice, 2000 L3 in 30 μL PBS/Pen-Strep were injected s.c. into the hind footpad.

### 2.3. E/S Production

For E/S production, *S. ratti* L3 were initially prepared as described above and then washed an additional six times in 10 mL HBSS supplemented with gentamycin (10%) and penicillin/streptavidin (100 U/mL) at 37 °C. L3 were transferred into a fresh sterile 15 mL tube and washed another six times as described above. Afterwards, L3 were suspended in 10 mL HBSS supplemented with gentamycin (10%), penicillin/streptavidin (100 U/mL) and also amphotericin (2.5 µg/mL) and incubated for 20 min at 37 °C. L3 were washed a final six times in HBSS supplemented with gentamycin (10%), penicillin/streptavidin (100 U/mL) and amphotericin (2.5 µg/mL). L3 were cultivated at 1000 L3/mL in RPMI 1640 supplemented with gentamycin (10%), penicillin/streptavidin (100 U/mL), saccharose (1%) and HEPES (10 mM) in 24-well culture plates at 37 °C and 5% CO_2_. Supernatant (SN) was collected every other day by collecting and replacing 500 µL medium per well over a period of 3 weeks. Pooled SN samples were filtered via a 0.2 µm filter, buffer-exchanged to PBS and concentrated to 500–1000 μg/mL using a 5 kDa cut-off spin column (Sartorius, Göttingen, Germany), and stored at −20 °C until use.

### 2.4. Cell Preparation

All cell preparation was performed as described in ref. [29] with minor adaptations.

Mesenteric lymph nodes (mLNs): mLNs were minced between the frosted ends of two glass slides in PBS to prepare single cell solutions. The cells were centrifuged (300× *g*, 5 min, 4 °C), resuspended in PBS and counted with Trypan Blue.

Lung cells: Lungs were harvested, cut into small pieces and placed in a 15 mL centrifuge tube with 2 mL RPMI1640 medium with Liberase TL (5 µg/mL) and DNase I (100 µg/mL) or with Dispase (0.4 U/mL), Collagenase VIII from *Clostridium histolyticum* (1 mg/mL) and DNase I (100 mg/mL). Lungs were shaken at 37 °C and 180 rpm. After 30 min incubation lungs were pipetted up and down with a 5 mL glass pipette, followed by a further 30 min incubation. The remaining tissue was pressed through a 70 µm cell strainer using a plunger of a 2 mL syringe. The strainer was rinsed with 10 mL cold PBS. Cells were centrifuged at 400× *g* for 5 min at 4 °C and erythrocytes were lysed with ACK lysis buffer and washed with PBS.

SI LPL: For isolation of lamina propria associated lymphocytes (LPLs) from the small intestine (SI), SI was excised below the stomach and above the cecum. SI tissues were placed on a PBS-soaked paper cloth. Fat was carefully removed with forceps and the intestines were opened longitudinally. To remove feces the SI was vigorously shaken in a Petri dish with water. Mucus was gently squeezed out with forceps followed by further washing in a Petri dish. The intestine was cut into 1 cm pieces (excluding the proximal 2 cm) and transferred into a 50 mL tube with 25 mL of cold HBSS with 10% FBS and vortexed. Each mouse was processed individually, and samples were kept on ice for less than 1 h. Samples were poured over a nitex mesh, placed in a 50 mL falcon and washed with pre-warmed HBSS. Tissue pieces were transferred with a forceps into a 50 mL tube with 15 mL HBSS containing 2 mM EDTA at 37 °C and vortexed. After 12 min incubation at 37 °C in an orbital shaker (220 rpm), cells were vortexed again. After washing with pre-warmed HBSS over the nitex mesh, tissue was again placed in EDTA/HBSS. The washing and EDTA incubation cycle, which included vigorous vortexing before and after the EDTA incubation, was repeated 2–3 times. Intestinal pieces were then digested in RPMI-1640 with 62.5 U collagenase VIII, 10% FBS for 15 min at 37 °C. After 10 min incubation the tubes were shaken manually, followed by further 5 min incubation. Digestion was stopped by adding 3.5 mL ice-cold RPMI-1640 with 5% FBS. The suspension was filtered first through a 100 µm strainer, followed by a 40 µm cell strainer placed in 50 mL tubes. The digested cells were centrifuged at 300× *g* for 10 min at 4 °C and washed with ice-cold RPMI-1640. All steps were completed within 3–3.5 h post-euthanasia.

### 2.5. Flow Cytometry

Flow cytometric analysis was performed as described previously, with minor alterations [29]. Briefly, 2–3 × 10^6^ cells were incubated with Zombie Yellow in 1 mL PBS for 20 min at 4 °C. Antibodies for surface staining were diluted in 50 µL of Fc block per sample, incubated for 30 min at 4 °C in flow cytometry tubes, and then washed to remove unbound antibodies. For surface staining only cells were fixed with 200 µL BD Fix. For the analysis of intracellular markers, the cells were fixed with either Foxp3 Fix/Perm or with 2% Formaldehyde in PBS, followed by subsequent permeabilization with Foxp3 Perm/Wash Buffer according to the manufacturer’s instructions. Cells were either stained for 30 min or overnight at 4 °C. Cells were measured on a five-laser Cytek Aurora and analyzed by FlowJo 10.7 and 10.10.

### 2.6. Culture and Stimulation of BM-Differentiated ILC2s

Culture and stimulation of BM-differentiated ILC2s were performed as described previously, with minor alterations [30,31]. Briefly, the femur and tibia of mice were stripped of muscle tissue and cut at both ends. The BM was then rinsed with cold PBS using a 21 G needle and collected in 20 mL ice-cold PBS. After lysis of the erythrocytes, the BM cells were washed three times with PBS and filtered through a 70 μm cell strainer. 5 × 10^5^ BM cells were incubated in 96-well round bottom plates in 200 µL RPMI 1640 medium with 10% FBS, 5 U/mL penicillin, 5 μg/mL streptomycin and 2 mM L-glutamine, supplemented where indicated with 10 ng/mL IL-2 and 10 ng/mL IL-7 in triplicates. For stimulation of ILC2, IL-33 (1 ng/mL) was added, in the presence or absence of 1 μg/mL E/S. After 5 days of incubation at 37 °C and 5% CO_2_, cytokines in the SN were quantified by ELISA according to the manufactures recommendations R&D Systems for IL-13 and IL-5 and Invitrogen for IL-9.

### 2.7. Statistical Analysis

Group comparisons were conducted using the following methods: unpaired Student’s *t*-test for parametric analysis of two groups at a single time point or condition; one-way ANOVA for parametric analysis of more than two unpaired groups followed by Bonferroni post-test using Graph Pad Prism 10.

## 3. Results and Discussion

### 3.1. CD4^+^ T Cells and ILC2s Produce IL-9 During S. ratti Infection

**Figure 1:**
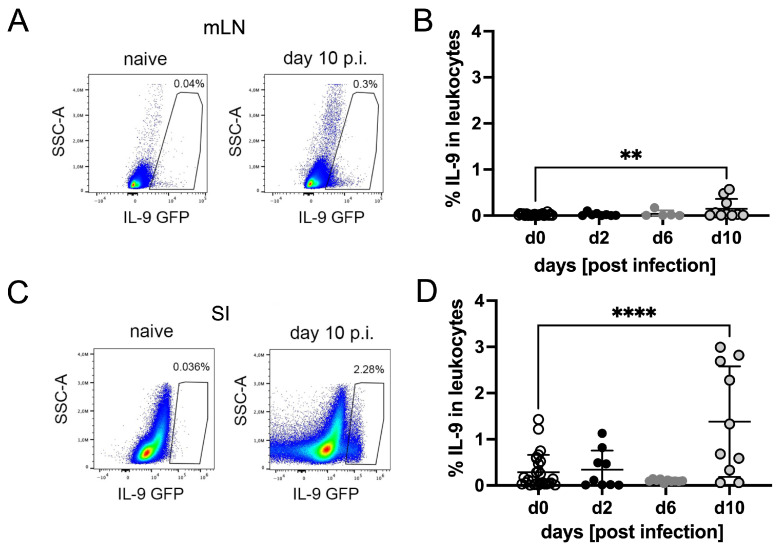
Dynamic of IL-9-producing leukocytes during the course of *S. ratti* infection. INFER mice were infected with 2000 *S. ratti* iL3 s.c. Mice were sacrificed at the indicated time points and mLNs (**A**,**B**) and SI-derived lamina propria cells (**C**,**D**) were isolated, stained and measured on a Cytek Aurora flow cytometer. Dot blots (**A**,**C**) show the expression of IL-9 GFP in CD45^+^, single and live leukocytes. Graphs show the expression of IL-9 GFP in mLN- (**B**) or SI (**D**)-derived leukocytes at indicated time points. The gating strategy is shown in Supplementary Figure S1. Each symbol represents an individual mouse; combined results from two experiments per time point with 3–6 mice per experiment and time point are shown. Asterisks indicate statistically significant differences in the mean, lines show the mean and error bars show SD (one-way Anova with Bonferroni post-test; ** *p* < 0.01, **** *p* < 0.0001).

To identify the dynamics of IL-9 production, as well as the relevant cellular sources of IL-9 during *S. ratti* infection, we used the “Interleukin Nine Fluorescent Reporter” (INFER) mouse model [22]. Here, the coding sequence of green fluorescent protein (GFP) is knocked in after the Il9 stop codon, under the control of the endogenous IL-9 promoter, thus allowing for detection of IL-9-expressing cells ex vivo in real time. A kinetic analysis of all IL-9-producing cells in the mesenteric lymph nodes (mLNs) and Lamina Propria Associated Lymphocytes (LPLs) derived from the SI during the course of *S. ratti* infection did not show a significant expansion before day 10 p.i. (Figure 1). At day 10 p.i. up to 3% of SI-derived LPLs produced IL-9, displaying high variation within the individual mice.

**Figure 2:**
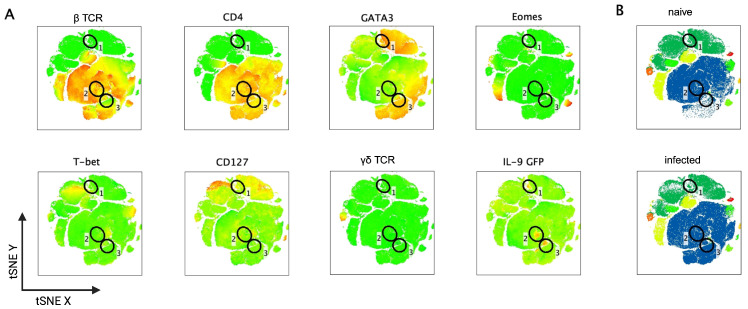
High-dimensional flow cytometric analysis of IL-9-producing cells in *S. ratti*-infected mice. INFER mice were infected with 2000 *S. ratti* iL3 or left naïve. Lamina propria cells from the SI were isolated at day 10 p.i. Cells were stained and measured by flow cytometry and analyzed by FlowJo Software. Cells were pre-gated as follows: CD45^+^ singlets, live cells, CD90.2^+^ lymphocytes. The gating strategy is shown in Supplementary Figure S1. Shown are representative results from one experiment with three naïve and three infected mice. Each sample was down-sampled to 35,000 events, and t-SNE (**A**) and FlowSom (**B**) Analysis was performed from the concatenated data. (**A**) Expression of βTCR, CD4, GATA3, EOMES, T-bet, CD127, γδ TCR and IL-9 GFP is shown for the combined t-SNE data. (**B**): The FlowSOM overlay from the t-SNE data for naïve and day 10 infected mice is shown. The different colours indicate different cell clusters according to the marker shown in (**A**).

An unbiased tSNE analysis of flow cytometry data from SI-derived LPLs of naïve and day 10 *S. ratti*-infected mice (Figure 2A) revealed three distinct IL-9-producing cell populations. One population of IL-9-producing cells overlapped with a CD127/GATA3-positive population (gate 1) but was negative for all other lineage markers and thus very likely represents ILC2s. Two populations (gate 2 and gate 3) overlapped with the βTCR- and CD4^+^ cell population; the IL-9-producing cells in gate 3 were GATA3^+^ and therefore may represent classical T helper 2 cells. By contrast, the IL-9-producing cells in gate 2 were GATA3^low^ and could principally represent Th9 cells, a specialized T helper cell subset that was described to develop in response to TGF-ß and IL-4 and is characterized by several additional transcription factors such as PU.1 and IRF4 while being GATA3^low^ [32,33,34,35]. The majority of the GATA3 and CD4 double-positive Th2 cells did not produce IL-9 (Figure 2A). The direct comparison of the SI-derived cell populations in naïve and *S. ratti*-infected mice showed a visible expansion of the entire CD4^+^ T cell compartment in infected mice only (Figure 2B, blue), while the three IL-9-producing cell populations were not visibly modulated (Figure 2B gates 1–3).

**Figure 3:**
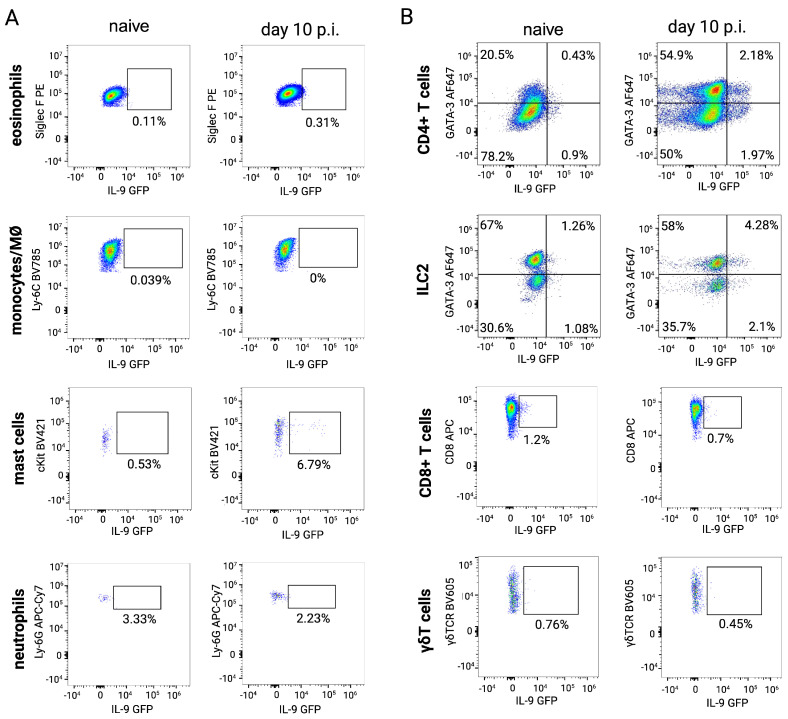
Analysis of IL-9-expressing cell types in the SI of *S. ratti*-infected mice. INFER mice were s.c. infected with 2000 *S. ratti* L3 or left uninfected. Lamina propria cells from the SI were isolated at day 10 p.i. and cells were either stained for (**A**) myeloid subsets (gating strategy is shown in Supplementary Figure S1) or (**B**) lymphoid subsets (gating strategy is shown in Supplementary Figure S2). All cells were pre-gated on CD45^+^ live leukocytes/lymphocytes. (**A**) Representative dot blots show the expression of IL-9 GFP in Siglec F^+^ eosinophils, Ly6C^+^ monocytes, FcεR^+^ cKit^+^ mast cells, and Ly6C^+^Ly6G^+^ neutrophils. (**B**) Representative dot blots show the expression of IL-9 GFP in CD8^+^ T cells, CD4^+^ T cells, CD127^+^ ILCs and γδ T cells.

As the fraction of IL-9-producing cells within the SI was very low (1–3%, Figure 1B, we next assessed IL-9 reporter expression within different gated immune cell populations (Figure 3). In line with the results presented in Figure 2A, the dominant IL-9-producing cell populations in the SI of day 10 *S. ratti*-infected mice were CD4^+^ T cells and ILC2s. In addition, intestinal mast cells also produced IL-9, as described in models of food allergy [36]. Eosinophils, monocytes/macrophages, neutrophils, CD8^+^ T cells and γδ T cells were negative for IL-9 in naïve and day 10 *S. ratti*-infected mice.

### 3.2. S. ratti Infection Interferes with the Expansion of IL-9-Producing ILC2s

**Figure 4:**
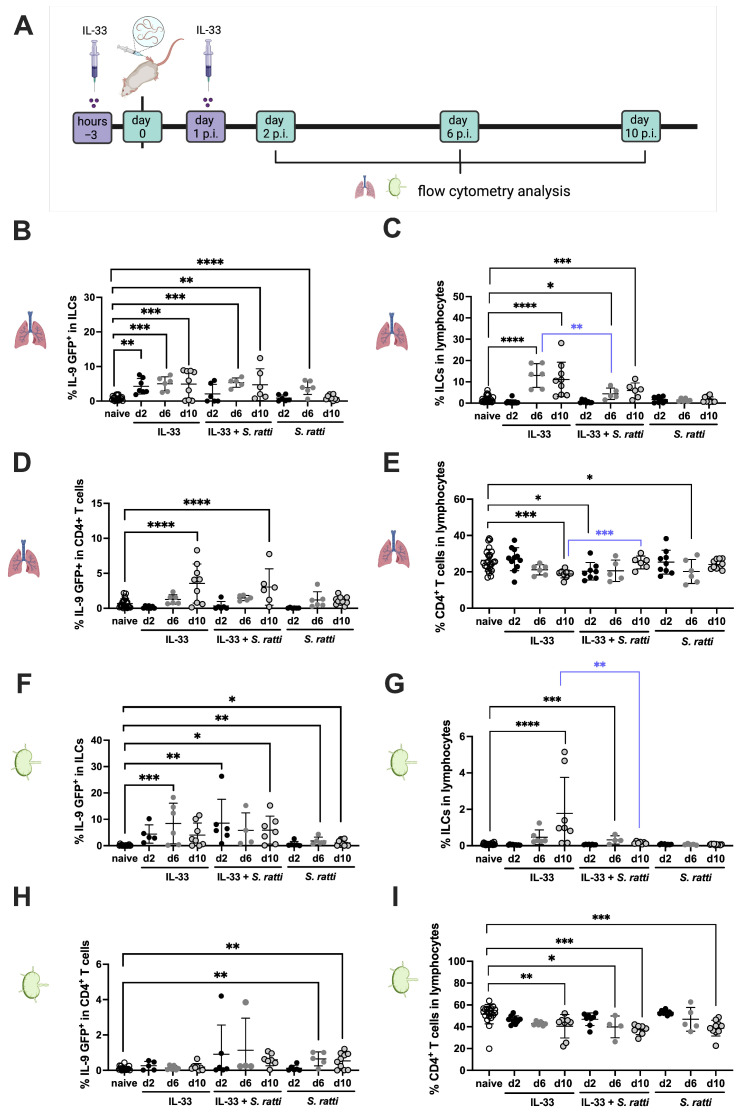
Dynamics of IL-9-producing T cells and ILC2s in IL-33-treated and *S. ratti*-infected mice. (**A**) Experimental set up: INFER mice were treated i.p. with 1 μg of rec. IL-33 3 h before and 24 h post *S. ratti* infection. Additionally, mice were infected with 2000 *S. ratti* iL3 or left uninfected. Lungs (**B**–**E**) and mLN cells (**F**–**I**) were isolated at days 2, 6 and 10 post-infection. Cells were stained and ILC2s were defined as CD45^+^ CD90.2^+^ βTCR^−^ CD49b^−^ γδTCR^−^ and CD127^+^ cells. CD4^+^ T cells were defined as CD45^+^ CD90.2^+^ βTCR^+^ and CD4^+^ lymphocytes. The statistical analysis shows the expression of IL-9 GFP in ILCs ((**B**) for lungs, (**F**) for mLNs) and CD4^+^ T cells ((**D**) for lungs, (**H**) for mLNs) and the frequency of ILCs ((**C**) for lungs, (**G**) for MLNs) and CD4^+^ T cells ((**D**) for lungs, (**I**) for mLNs) within all lymphocytes at the indicated time points. Every symbol represents an individual mouse; lines show the mean and error bars SD. Combined results from two (days 2 and 6) or three (day 10) experiments are shown. Asterisks in black indicate statistically significant differences in the mean compared to naïve mice. Asterisks in blue indicate statistically significant differences in the mean between IL-33-treated and IL-33-treated + *S. ratti*-infected mice at day 6 or day 10 (one-way Anova with Bonferroni post-test; * *p* < 0.05, ** *p* < 0.01, *** *p* < 0.001, **** *p* < 0.0001).

While the intestinal parasite burden peaks at day 6 post *S. ratti* infection, and reduces thereafter [28], expansion of IL-9-producing cells in the mLNs and SI of *S. ratti*-infected mice was only apparent by day 10 p.i., and not before (Figure 1). In light of the central role that IL-9 plays in the mast cell-mediated ejection of *S. ratti* from the SI [12], we assessed whether the IL-9 production and/or expansion of the IL-9-producing cells may be antagonized and thus delayed by the *S. ratti* parasites. To test this hypothesis, we provoked IL-9 production by ILC2s and CD4^+^ T cells in vivo by application of recombinant (rec.) IL-33 as described previously [37], and compared IL-33-induced responses in naïve or *S. ratti*-infected mice (Figure 4A). IL-33 treatment induced IL-9 production by ILC2 from day 2 to day 10 posttreatment and expanded the ILC2 population from day 6 to day 10 post-treatment (Figure 4B,C, black asterisks). *S. ratti* infection in addition to IL-33 treatment resulted in a significant reduction in the total percentage of ILC2s within total lung lymphocytes at day 6 p.i. (Figure 4C, blue asterisks). Of note, this suppression was visible despite the high intraexperimental variation with the individual mice.

IL-33 treatment also induced an expansion of the percentage of IL-9-producing T cells within the CD4^+^ T cells in the lung by day 10 post-treatment (Figure 4D) but did not increase the percentage of all CD4^+^ T cells (Figure 4E). Neither the percentage of IL-9-producing CD4^+^ T cells nor the frequency of CD4^+^ T cells within the lungs of IL-33-treated mice were reduced by additional *S. ratti* infection (Figure 4D,E). By contrast, CD4^+^ T cell frequencies at day 10 post IL-33 treatment increased in mice that were additionally infected with *S. ratti* (Figure 4E, blue asterisks).

The frequency of IL-9-producing ILC2s within total mLN ILC2s, induced by IL-33 treatment, was not affected by additional *S. ratti* infection (Figure 4F). However, the frequency of total ILC2s within the mLN cells that expanded by day 10 post IL-33 treatment was significantly reduced by additional *S. ratti* infection (Figure 4G, blue asterisk). *S. ratti* infection alone also increased the percentage of IL-9-producing ILC2s in the mLNs at days 6 and 10 p.i. (Figure 4F) but did not expand the ILC2 population within all mLN lymphocytes (Figure 4G). MLN-derived CD4^+^ T cells, by contrast, produced IL-9 selectively in response to *S. ratti* infection by day 6 to day 10 p.i. (Figure 4H), while the overall percentage of CD4^+^ T cells in mLNs of day 10 *S. ratti*-infected mice was reduced compared to those of naïve mice (Figure 4I). As IL-33 treatment did not induce IL-9 in mLN-derived CD4^+^ T cells (Figure 4H), it was not possible to test a putative suppression of T cell-derived IL-9 by *S. ratti* infection in this lymphatic organ.

It was also not possible to perform these analyses with SI-derived LPLs because the IL-33 treatment alone drastically reduced their viability (Supplementary Figure S3). Thereby, selectively the LPL preparations from mice that were treated with IL-33 displayed impaired viability, while LPL preparations from the SI of either naïve mice, *S. ratti*-infected mice, or even mice that were treated with IL-33 but additionally infected with *S. ratti* yielded viable cells in the majority of samples.

**Figure 5:**
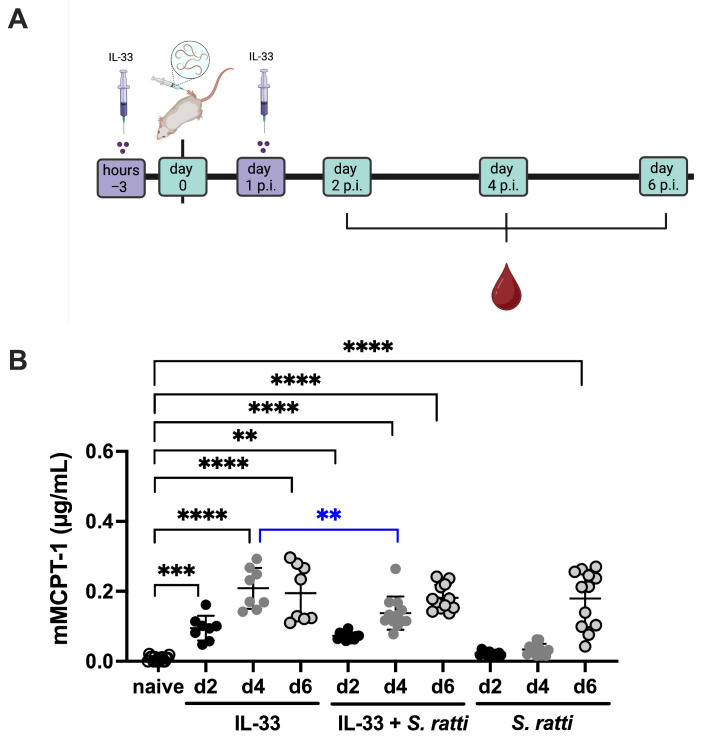
Dynamics of mucosal mast cell activation in IL-33-treated and *S. ratti*-infected mice. (**A**) Experimental set up: BALB/c mice were treated i.p. with 1 μg of IL-33 3 h before and 24 h post *S. ratti* infection. Additionally, mice were infected with 2000 *S. ratti* iL3 or left uninfected. Blood was drawn at days 2, 4 and 6 p.i. (**B**) mMCPT-1 was quantified in the serum by ELISA. Shown are the combined results from two independent experiments with 3–5 mice per experiment and time point. Every symbol represents an individual mouse; lines show the mean and error bars SD. Asterisks in black indicate statistically significant differences in the means compared to naïve mice. Asterisks in blue indicate statistically significant differences in the mean between IL-33-treated and IL-33-treated + *S. ratti*-infected mice. Non-significant differences are not indicated (one-way Anova with Bonferroni post-test; ** *p* < 0.01, *** *p* < 0.001, **** *p* < 0.0001).

In summary, these results show that *S. ratti* infection did not modulate IL-33-mediated IL-9 production by CD4^+^ T cells in the lung but specifically impaired expansion of ILC2s, which are a major source of IL-9 in lungs and mLNs. As IL-9 was shown to promote mast cell activation and expansion during intestinal helminth infection [21] and the early degranulation of mucosal mast cells specifically during *S. ratti* infection depends on IL-9 [12,24], we tested if this suppression of the IL-33-induced expansion of ILC2s as major source of IL-9 would also suppress mucosal mast cell degranulation. To this end, we quantified mucosal mast degranulation by measuring the concentration of mMCPT-1 [38] in the serum of the IL-33-treated group compared to the IL-33-treated and *S. ratti*-infected group and the *S. ratti*-only-infected group (Figure 5A). Mucosal mast cell degranulation was rapidly induced 2 days after IL-33 treatment (Figure 5B, black asterisks). Additional infection with *S. ratti* resulted in a significant reduction in this IL-33-induced mast cell degranulation at day 4 post-treatment/infection (Figure 5B, blue asterisks). Compared to naïve mice, *S. ratti* infection alone triggered significant mast cell activation only by day 6 p.i. (Figure 5B, black asterisks). These results suggest that the *S. ratti* infection-mediated reduction in the percentage of IL-9-producing ILC2 that are induced by IL-33 treatment is translated into reduced mucosal mast cell activation, i.e., promotes parasite establishment in the SI.

### 3.3. S. ratti E/S Products Interfere with IL-33-Induced IL-9 and IL-13 Secretion by BM-Derived ILC2 Cultures

**Figure 6:**
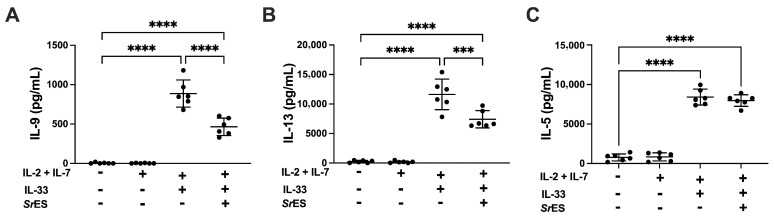
IL-33-stimulation of BM ILC2s. Bone marrow cells from C57BL/6 mice were isolated and cultivated at 5 × 10^5^ cells/well with IL-2 (10 ng/mL), IL-7 (10 ng/mL) in the presence or absence of IL-33 (1 ng/mL) for 5 days in 96-well round-bottom culture plates in triplicates. 1 µg/mL *S. ratti* derived ES products (*Sr*ES) was added in indicated groups. Concentrations of IL-9 (**A**), IL-13 (**B**) and IL-5 (**C**) in the culture supernatant were quantified by ELISA. Shown are combined data from two independent experiments; lines show the mean and error bars SD. Asterisk in green indicates statistically significant differences in the mean. (one-way Anova with Bonferroni post-test *** *p* < 0.001, **** *p* < 0.0001).

Helminths have been shown to downmodulate their host’s immune response via excretory secretory products (E/S) [39]. For instance, the intestinal parasite *Heligmosomoides polygyrus* secretes proteins that induce expansion of Treg [40,41] or antagonize IL-33 function [42]. To directly investigate a potential impact of *S. ratti*-derived E/S proteins on ILC2s, we cultured bone marrow (BM) cells with IL-2 and IL-7 to support ILC2 differentiation and with IL-33 to activate ILC2s within the culture as described [30,31], in the presence or absence of *S. ratti* E/S. It should be noted that this experimental approach did not aim at using highly purified, sorted ILC2s, as previous work showed that the IL-13 producing cell population in these BM-cell cultures represents lin^−^CD45^+^ICOS^+^ ILC2s [30,31]. IL-9, IL-13 and IL-5 were quantified in the culture supernatants (SN) (Figure 6). The addition of *S. ratti* E/S reduced the total amount of IL-9 and IL-13 in the SN by around 50% (Figure 6A,B), while IL-5 production was not modulated (Figure 6C). Although we cannot distinguish if the reduced IL-9 and IL-13 production was due to impaired IL-33-mediated ILC2 expansion and/or impaired production of these cytokines by the individual ILC2, the net effect results in a similar reduction in IL-9 levels that we recorded in vivo in IL-33-treated/*S. ratti*-infected mice (Figure 4). The interesting observation that IL-5 production by BM-derived ILC2 was not affected by *S. ratti* E/S products may suggest a polarization of ILC2 function by *S. ratti*-derived E/S that is under current investigation.

Taken together, our study shows that both ILC2s and CD4^+^ T cells represented dominant IL-9-producing cell populations during murine *S. ratti* infection. Therefore, our experimental approach did not directly reveal if T cell- or ILC2-derived IL-9 was more relevant for host defence. In this context, specifically IL-9-producing T cells were shown to be protective in *N. brasiliensis* infection, as adoptive transfer of Th9 cells into RAG KO mice reduced intestinal parasite burden [22]. Likewise, Th9 cells were described to expand during human *S. stercoralis* infection [43]. On the other hand, using the alarmin IL-33 as an external stimulator of IL-9 production and subsequent mucosal mast cell degranulation, we observed that IL-33 treatment also reduced intestinal parasite burden in RAG KO mice (i.e., in the absence of T cells), but not in RAG *γ* c KO mice (i.e., in the additional absence of all ILC) [37]. Using the INFER IL-9 reporter mouse, we here show that *S. ratti* infection downmodulated the total percentage of ILC2s in lungs and mLNs of IL-33-treated mice. IL-33 activated lung CD4^+^ T cells, by contrast, were not affected by additional *S. ratti* infection. These findings suggest that *S. ratti* predominantly targets the IL-9-producing ILC2 population and not the IL-9-producing T cell population to suppress IL-9 production via suppression of ILC2 expansion in vivo and thereby delay its mast cell-derived ejection from the intestine. While this also suggests that ILC2-derived IL-9 may be more important in anti-*S. ratti* immunity, the exact relevance of T- and ILC2-derived IL-9 in the ejection of intestinal parasites still needs to be evaluated, using cell type-specific cytokine KO mice.

Our findings agree with earlier studies showing that *S. ratti* delayed its ejection from the SI by expansion of Foxp3^+^ Treg and by induction of negative checkpoint receptors on CD4^+^ T cells (reviewed in [3]). Depletion of either Treg or deficiency for one checkpoint receptor, B and T lymphocyte attenuator, or its ligand, Herpes virus entry mediator, reduced intestinal parasite burden in the context of accelerated mucosal mast cell activation and increased in vitro IL-9 production by mLN cells [24,25]. While these studies provided a direct causal link between elevated IL-9, increased mucosal mast cell degranulation, and reduced parasite burden, the modulated IL-9-producing cell populations were not identified.

We extend the repertoire of *S. ratti*-induced immunomodulation by the observation that *S. ratti*-derived E/S products interfere with IL-9 and IL-13 production by BM-derived ILC2s in vitro, thus suggesting an additional immune modulatory pathway that directly supresses ILC2-derived IL-9. In light of the multiple immunomodulatory pathways used by *S. ratti* to downmodulate the IL-9-driven intestinal inflammation, it is tempting to speculate that this immunosuppression contributed to the improved viability of SI-derived LPL in IL-33-treated and *S. ratti*-infected mice compared to IL-33-treated mice.

## Data Availability

The original contributions presented in this study are included in the article/Supplementary Material. Further inquiries can be directed to the corresponding author.

## Supplementary Materials

The following supporting information can be downloaded at https://www.mdpi.com/article/10.3390/pathogens15030257/s1, Table S1: key resource table. Figure S1: Gating strategy for myeloid cells from SI; Figure S2: Gating strategy for ILCs and T cells; Figure S3: Viability of SI-derived LPL.

## Abbreviations

The following abbreviations are used in this manuscript:

BM	bone marrow
ILC2	group 2 innate lymphoid cell
INFER	Interleukin Nine Fluorescent Reporter
mLN	mesenteric lymph node
p.i.	post-infection
mMCPT1	mouse mast cell protease-1
SI	small intestine
