# Supplementary material for: Dynamics of Interleukin-9 Producing Lymphocytes in Strongyloides ratti-Infected Mice
Source: Pathogens. 2026 Feb 28;15(3):257. doi: 10.3390/pathogens15030257 (PMC13029138; doi:10.3390/pathogens15030257)
Supplement: Supplementary file 1 [file pathogens-15-00257-s001.zip › pathogens-4087018-supplementary.pdf]

Supplementary Material

Table S1: key resource table

REAGENT or RESOURCE	SOURCE	IDENTIFIER
**Antibodies (anti-mouse)**		
CD19 (PE/Dazzle)	BioLegend	Cat# 100495, RRID: AB_2564001
CD19 (Biotin)	BioLegend	Cat# 115504, RRID: AB_ 313639
CD4 (Brilliant Violet 510™)	BioLegend	Cat# 100559, RRID:AB_ 2562608
CD4 (BUV395)	BD Bioscience	Cat# 563790, RRID:AB_2738426
CD45 (Alexa Fluor® 700)	BioLegend	Cat# 147716, RRID:AB_2750449
CD49b (PE/Cy7)	BioLegend	Cat# 108922, RRID: AB_2561460
CD49b (PE/Cy7)	BioLegend	Cat# 103518, RRID: AB_ 2566103
CD90.2 (APC Fire750)	BioLegend	Cat# 105348, RRID:AB_2800563
CD11b (Brilliant Violet 650™)	BioLegend	Cat# 101259, RRID:AB_2566568
CD11c (Biotin)	Biolegend	Cat# 117304, RRID:AB_313773
CD127 (Brilliant Violet 785™)	BioLegend	Cat# 135037, RRID:AB_2565269
CD170/Siglec-F (PE)	BioLegend	Cat# 155506, RRID:AB_2750235
CD335/NKp46 (PE)	BioLegend	Cat# 137604, RRID:AB_ 2235755
c-Kit (Brilliant Violet 421™)	BioLegend	Cat# 135124, RRID:AB_2562237
Eomes (BD Horizon™ PE-CF594)	BD Bioscience	Cat# 567167, RRID: AB_ 2916484
FcεRIa (APC)	BioLegend	Cat# 134316, RRID:AB_ 10640121
FcεRII (PE)	BioLegend	Cat# 134307, RRID:AB_1626104
GATA3 (Alexa Fluor® 647)	BioLegend	Cat# 653810, RRID: AB_ 2563217
GFP (Alexa Fluor® 647)	BioLegend	Cat# 338008, RRID: AB_ 2563288
NK1.1 (PerCP/Cyanine5.5)	BioLegend	Cat# 156526, RRID:AB_2894655
Ly-6C (BV785)	BioLegend	Cat# 128041, RRID:AB_ 2565852
Ly-6G (APC/Cyanine 7)	BioLegend	Cat# 127624, RRID:AB_ 10640819
T-bet (Pacific Blue ™)	BioLegend	Cat# 644808, RRID: AB_ 1595479
TCRb (Brilliant Violet 711™)	BioLegend	Cat# 109243, RRID:AB_ 2629564
TCRg/d (Brilliant Violet 605™)	Biolegend	Cat# 118129, RRID:AB_ 2563356
Zombie Yellow™ Fixable Via-	BioLegend	Cat# 423103
Experimental models: Organisms/strains		

mouse: BALB/c	Charles River	N/A
mouse: INFER<br>mouse: C57BL/6<br>Rats: Wistar<br><br>*Strongyloides ratti*	In-house breeding<br>Charles River<br>In-house breeding	
Critical commercial assays		
Mouse IL-9 Uncoated ELISA	Thermo Fisher Scientific	Cat# 88-8092-88, RRID:AB_2575179
Mouse IL-13 DuoSet ELISA	R&D Systems	Cat# DY413-05
Mouse IL-5 DuoSet ELISA	R&D Systems	Cat# DY405-05
Mouse mast cell protease 1	Thermo Fisher Scientific	Cat# 88-7503-88
Chemicals, peptides, and recombinant proteins		
Amphotericin	Gibco	Cat# 15290026
Charcoal	Roth	Cat# 0998.3
Collagenase	Sigma-Aldrich	Cat# C2139-1G
Dispase	Sigma-Aldrich	Cat# D4693-1G
DNase I	Roche	Cat# 4536282001
Fecal bovine serum (FBS)	Capricorn	Cat# FBS-11A
Gentamycin	Capricorn	Cat# GEN-10B
Hanks' Balanced Salt Solution	Capricorn	Cat# HBSS-3A
HEPES	Capricorn	Cat# HEP-B
Liberase TL	Roche	Cat# 05401020001
Penicilin/Streptomycin	Capricorn	Cat# PS-B
Recombinant Mouse IL-2	BioLegend	Cat# 575406
Recombinant Mouse IL-7	BioLegend	Cat# 577806
Recombinant Mouse IL-33	BioLegend	Cat# 580506
ROTI Fair 10x PBS	Roth	Cat# 1105.1
Roti Histofix Formaldehyde 4%	Roth	Cat# P087.6
RPMI-1640	Capricorn	Cat# RPMI-A
Software and algorithms		
FlowJo 10.10	Becton Dickinson & Company (BD)	
GraphPad Prism 10.6.1	GraphPad Software	

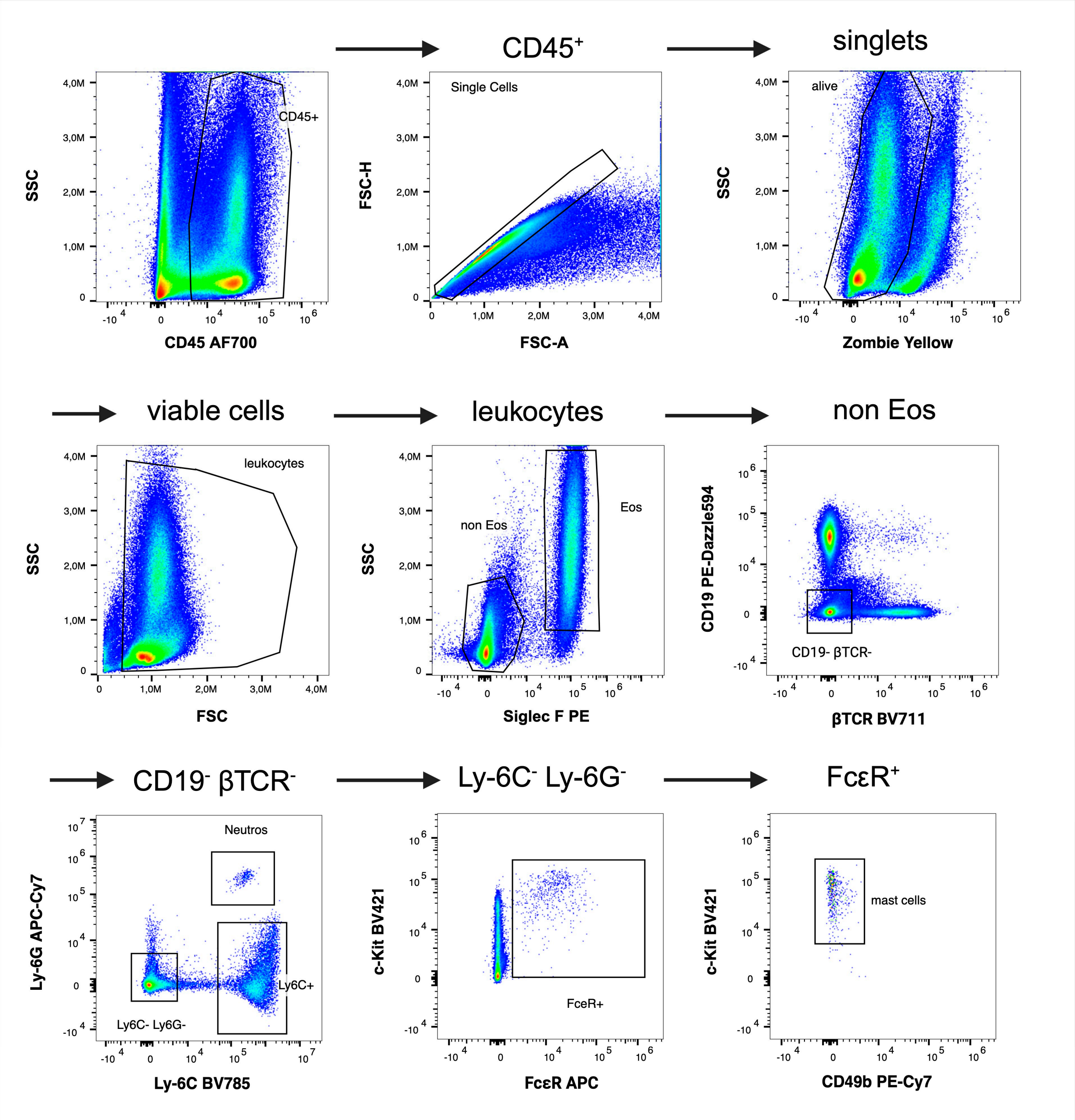

Supplementary figure S1: Gating strategy for myeloid cells from SI

Cells from the SI of day 10 *S. ratti*-infected mice were pregated as CD45+ cells, singlets, viable (Zombie negative) leukocytes. Siglec F+ cells were defined as eosinophils. From the SiglecF- cells CD19+ B cells and βTCR+ cells were excluded. In the non-B non-T cell gate Ly6C+Ly6G+ cells were defined as neutrophils, Ly6C+ as monocytes/macrophages and the Ly6C-Ly6G- cells were further analysed according to their expression of FcER. Mast cells were defined as FcER+ cKit+ mast cells.

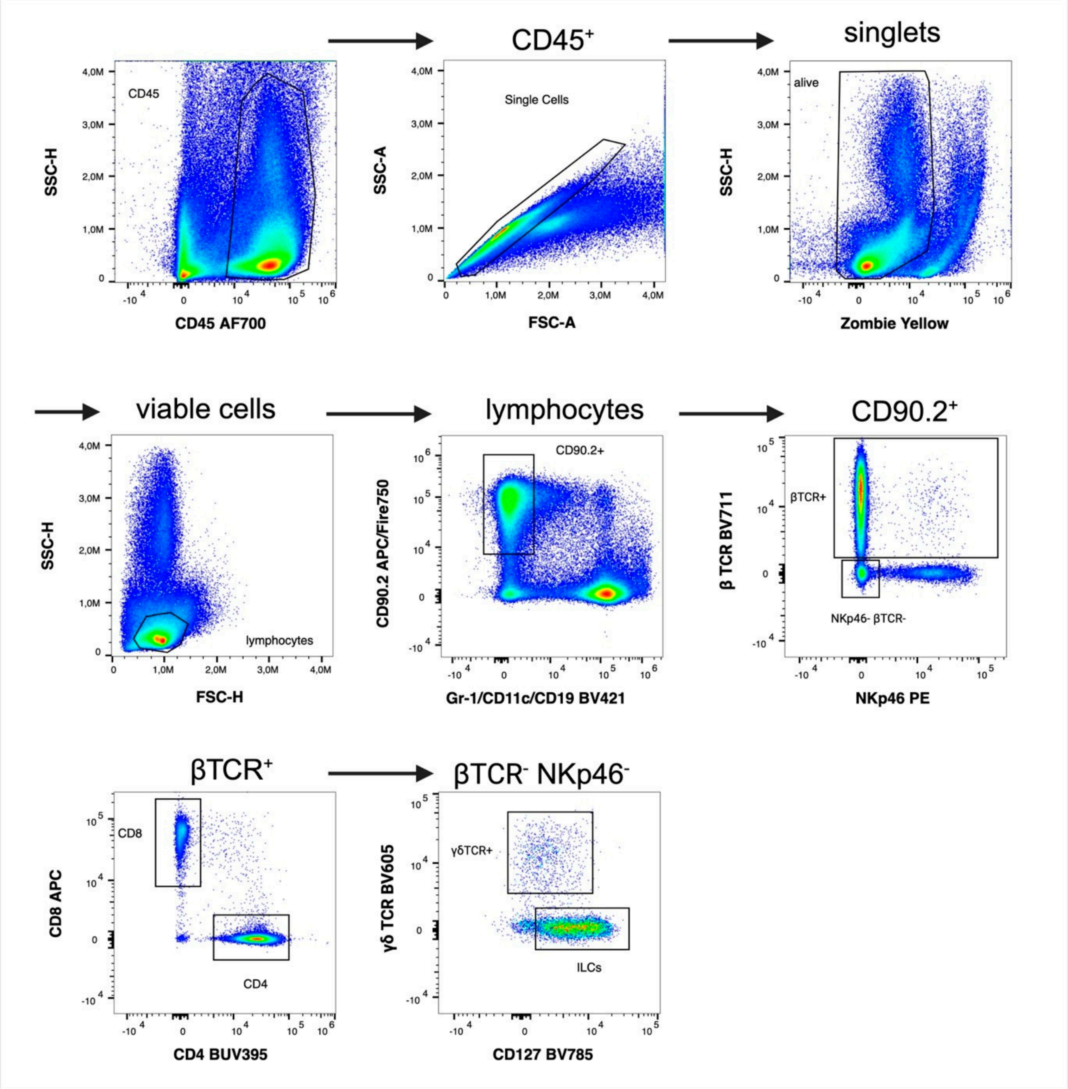

Supplementary figure S2: Gating strategy for ILCs and T cells.

Cells from the SI of day 10 *S. ratti*-infected mice were pregated as CD45^{+} cells, singlets, viable (Zombie negative) lymphocytes. Gr-1^{+}/CD11c^{+}/CD19^{+} cells were excluded. Cells expressing CD90.2 were further gated as followed: βTCR^{+} were further divided into CD4^{+} or CD8^{+} T cells depending on their CD4 or CD8 expression. βTCR^{-} CD49b^{-} were divided in γδT cells or CD127^{+} ILCs.

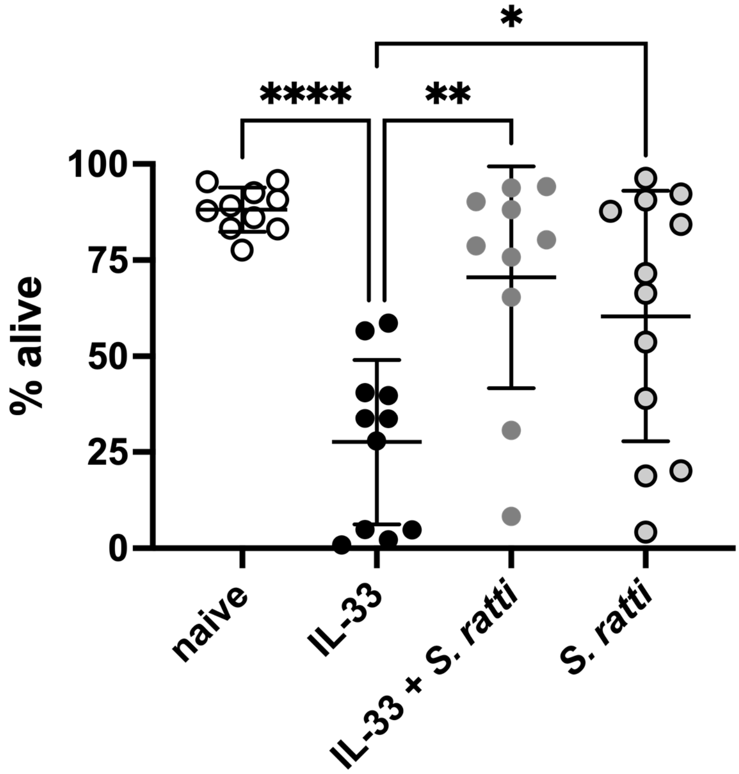

Supplementary figure S3: Viability of SI-derived LPL

INFER mice were treated i.p. with PBS or 1 µg of IL-33 3 h before and 24 h post *S. ratti* infection. Additionally, mice were infected with 2000 *S. ratti* iL3 or left uninfected. Lamina propria cells from the SI were isolated and stained for flow cytometry. A) The statistical analysis shows the percentage of Zombie UV negative cells (depicted as % alive) on the y- axis. The x-axis shows the 4 different groups. Each symbol represents an individual mouse, lines show the mean and error bars show SD. Combined results from 4 experiments with 1-4 mice per group are shown. Asterisks indicate statistically significant differences of the mean (one-way ANOVA with Bonferroni post test: * $p < 0.05$, ** $p < 0.01$, **** $p < 0.0001$).
